# No Time Like the Present: Centring Politics in the Global NCD Policy Agenda: Comment on "Barriers and Opportunities for WHO ‘Best Buys’ Non-Communicable Disease Policy Adoption and Implementation From a Political Economy Perspective: A Complexity Systematic Review"

**DOI:** 10.34172/ijhpm.9145

**Published:** 2025-06-17

**Authors:** Lana M. Elliott, Stephanie M. Topp

**Affiliations:** ^1^Centre for Justice, School of Public Health and Social Work, Queensland University of Technology, Brisbane, QLD, Australia.; ^2^College of Medicine and Dentistry, James Cook University, Townsville, QLD, Australia.

**Keywords:** Best Buys, Non-communicable Diseases, Policy, Political Economy

## Abstract

The World Health Organization’s (WHO’s) non-communicable disease (NCD) Best Buys provides a comprehensive package of technically sound policy advice in response to the growing global burden of NCDs. However, despite these policy mechanisms being touted as beneficial to countries’ health and economic bottom lines, uptake has remained slow and globally disparate. Loffreda and colleagues’ analysis draws attention to the importance of political economy forces in shaping governments’ responses to NCDs and, in particular, their uptake of the NCD Best Buys. In building on this work, we examine the history and instances of contemporary application of the NCD Best Buys to consider the limitations of these technocratically framed policy recommendations. In doing so, we highlight the risks present in focusing on the technical – while negating the contextually nuanced political – dimension of policy adoption. We thus advocate for greater political engagement in policy design and implementation as well as a revitalised "double-loop" approach to NCD policy learning, where policy and system feedback is not solely used to reify policy advice but rather interrogate the assumptions underpinning such.

## Introduction

 Irrespective of whether they are supported by robust science or not, policy decisions are inherently political. And in the lead up to the fourth United Nations High Level Meeting on Non-Communicable Diseases (NCDs), Loffreda and colleagues’^1^ political economy analysis of forces influencing state responses to NCDs is a timely reminder that effective policy-making requires so much more than sound technical evidence.

 Drawing together findings from 157 studies spanning all World Health Organization (WHO) regions and countries faced with various political and economic realities, Loffreda and colleagues’^1^ analysis clearly demonstrates how the uptake of seemingly universal policy recommendations is frequently hampered by complex local political realities. Although often framed as apolitical and inherently “good” health and economic policy, global NCD policy recommendations have rarely been sufficient to advance complex and contextually specific policy-making or to ensure that resulting policies are well-adapted to local realities. Loffreda and colleagues’^1^ work hence highlights the importance of concurrently foregrounding systems thinking, political economy analysis and considerations of implementation into the NCD policy-making landscape. Refocusing NCD policy research from the content—the “what” of NCD policies—to the processes and motivations—the “how” and “why”—is essential to support governments and other health interested parties bridge the persistent know-do gap critical to addressing the global rise in NCDs. By analysing the history of the NCD Best Buys and considering insights from our own work on NCD Best Buys-aligned policies, we argue here that there are significant risks to policy progress—and, by extension, population health—associated with an overly technocratic approach to NCD policy. Effective NCD policy action is contingent on the integration of politically (as well as technically) informed approaches to policy design and implementation as well as the application of far more effective policy feedback mechanisms capable assessing the very assumptions underpinning policy action.

## The History and Design of the NCD Best Buys

 The WHO NCD Best Buys have been a cornerstone of global NCD policy advice for 15 years. Drafted in preparation for the first United Nations High Level Meeting on NCDs in 2011,^2^ the Best Buys were originally based on the 4×4 conceptualisation of NCDs (namely, four major NCDs: cancer, cardiovascular disease, chronic obstructive pulmonary disease, and diabetes; and their four major modifiable risk factors: smoking, alcohol consumption, poor diet, and inadequate physical activity). Although the 4×4 model is widely seen as capturing the major contributors to the global rise in NCDs, it has been criticised for failing to reflect the complex realities and underpinning drivers of NCDs in low- and middle-income settings.^3^ Moreover, this very disease-specific approach to NCD prevention and control is, like other siloed programs, likely to constrain vital health systems strengthening reforms and considerations of cross sectoral domains, such as the commercial determinants of health.^4^

 The NCD Best Buys were largely drafted by health economists and health financing experts, with interventions selected for inclusion based on four criteria: (*i*) health impact; (*ii*) cost-effectiveness; (*iii*) cost of implementation; and (*iv*) feasibility of scale up, particularly in low-resource settings.^2^ The original 14—later expanded to 28—Best Buy policy recommendations are commonly framed as health and economic “win-wins,” supported by strong evidence of their potential to improve both health outcomes and economic performance in implementing countries. In this sense, the Best Buys are a product to their time: constructed on a robust technical evidentiary basis and, importantly, framed with an economic investment case, recognising the considerable power of economic arguments in mobilising multisectoral action.

 Yet explicit or implicit recognition of the political economy forces shaping both design and implementation phases of any NCD policy have been largely absent from the Best Buys. Early iterations of the Best Buys overlooked political considerations entirely, while more recent versions offer only limited acknowledgment—for example, single sentence footnotes such as “requires multisectoral action…,”—which suggest a continued underestimation of the political complexity involved in delivering such action.^2,5,6^

 The approach taken by authors and advocates of the Best Buys, in identifying and advocating for cost-effective NCD interventions thus largely rests on two assumptions: (*i*) that lack of awareness alone explains the failure to adopt these interventions, and (*ii*) that cost-effectiveness is a sufficient driver of policy action. Fifteen years since their inception, however, the relatively slow and disparate global uptake of these “win-win” policies—and even slower progress in reducing the NCD burden—suggests a more complex reality in translating policy aspiration into concrete actions and outcomes.

## The Ramifications of a Normative Approach to NCD Policy and Policy-Making

 Global and national efforts have often prioritised the identification and promotion of cost-effective, standardised interventions for responding to NCDs. However, comparatively little attention has been directed towards navigating the pragmatic or political challenges of implementing such reforms. As Herrick observes, “the clear rhetoric of evidentiary simplicity” that characterises much NCD related research and policy advice stands in stark contrast with “the exceptionally expansive co-constitutive and inter-dependent nature” of NCDs and their social, political and commercial determinants.^7^ In seeking to render the NCD agenda more tractable for policy actors, advice often over simplifies the complex and contested nature of health reform. This simplicity risks overlooking—or even undermining—the iterative, negotiated, and inherently political processes through which all health policy is developed and implemented. The pursuit of “win-win” solutions, without critical engagement with underlying power dynamics can lead to a narrow focus on whether recommended policy mechanisms are formally adopted, rather than examining how they emerged, whose interests they serve and, ultimately, whether what is implemented actually advances intended health outcomes.

 The notion of public policy as a neutral evidence-driven tool for solving societal problems—rather than a product of political or ideological contestation—holds strong appeal, as reflected in the enduring influence of the evidence-based policy movement.^8^ However, there are significant limitations in relying on such instrumental and often decontextualised policy prescriptions as the basis for robust implementation roadmaps, or at least predictable, policy outcomes. Loffreda and colleagues’ analysis reinforces growing evidence that the uptake of NCD Best Buy policy recommendations is neither linear nor assured, and rarely yields uniform outcomes across diverse health, economic, trade and commercial interests they implicate.^9^ Our own research analysing the political economy forces underpinning NCD-related tax policy in Vanuatu and Fiji reflects multiple and cross-cutting socio-political and cultural dynamics in both the appetite for adopting NCD Best Buy policy recommendations and subsequentimplementation.^10-12^ Rather than being the product of cohesive, goal-oriented government action, policy design and adoption in these settings were shaped by ambiguity around the interests and end goals of various actors. In fact, the absence of a shared understanding of the policy’s purpose or intended effects enabled diverse actors to claim the introduction of NCD-related taxes as sectoral “successes,” without the need for evidence of efficacy or impact through evaluation.^12^

## Way Forward

 Policy-making is not a mechanical process in which policies are instrumentally imposed on a context. Rather, policies and their implementation processes are products of – and windows into^13^ – context-specific, time-bound sociocultural and political dynamics. They are continually shaped and reshaped by the environments in which they are enacted. Just as the determinants of NCDs are complex and multifaceted, so too is policy-making itself a messy and iterative process shaped by evolving ideas, interests and institutions.

 As Peters and Bennett^14^ argue, the sustained pursuit of universal evidence for *what works *in global health policy and health systems—and currently exemplified in the Best Buys—can obscure equally and sometimes more pressing questions that must be answered by country-level policy-makers: *what will work for us, in our context? *And *how can we make this intervention work, based on our goals and interpretations?* The assumed universality of the NCD Best Buys, including their embedded assumptions about what motivates policy adoption and what outcomes will follow, risks overlooking contextual nuances that shape why and how policies are taken up – and to what end.

 We believe a revitalised approach to developing and refining NCD policy, with a renewed emphasis onplace-based *learning* is needed. Insights from organisational learning theory amplified in the Alliance for Health Policy and Systems Research’s flagship report on learning health systems offer a useful point of departure – with its distinction between single-loop and double-loop learning in health systems.^15^ Single-loop learning refers to a process where organisations adjust their actions or strategies based on feedback, but without questioning the underlying assumptions, goals, or values guiding those actions. Current global NCD policy exemplified in the WHO Best Buys could be argued to be based on this logic of single-loop learning: universal policies and recommendations are refined over time, and made available to newly “aware” policy-makers but with little attention to the underlying assumptions they contain or the diverse conditions and contexts into which they are being introduced. In contrast, double-loop learning involves questioning and revising the deeper assumptions, norms, and institutional logics that shape both policy and practice. Applying the logic of double-loop learning to NCD policy would support more context-sensitive approaches – shifting attention from technical refinement to critical engagement with the assumptions, values, and institutional dynamics that shape policy relevance and uptake.

 Evolving global NCD policy advice toward the principles of double-loop learning would mark a necessary shift – from refining “what works” to understanding *why*, *how*, and *for whom* Best Buy-style policies work in diverse settings. Political economy analysis offers a valuable methodological entry point for this work. For policy-makers and advocates alike, political economy analysis helps unpack how contextual factors—including power relations, institutional dynamics and ideological orientations—influence policy processes and outcomes. These insights can allow for the identification of context-specific leverage points that elevate issues and ideas onto policy agendas; framing arguments to maximise public or political support; and identifying potential policy traps capable of stalling progress, or indeed, coopting its intended purpose. Such analysis is especially relevant in a global political landscape that has become increasingly fragmented and contentious. In the past six months alone, the global health community has encountered heightened resistance, reflecting not technical disputes, but political and ideological tensions.

 Now more than ever navigating these complexities is essential. Whether in relation to NCDs or other pressing health challenges, the appeal of simplified, technical and apparently universal “solutions” must be resisted. Embracing complexity—and engaging directly with the politics of health and health systems—remains our best strategy for advancing equitable and effective policy, and for building more just and resilient health systems globally.

## Ethical issues

 Not applicable.

## Conflicts of interest

 Authors declare that they have no conflicts of interest.
